# Influence of simplified, higher-concentrated sodium ascorbate application protocols on bond strength of bleached enamel

**DOI:** 10.4317/jced.55153

**Published:** 2019-01-01

**Authors:** Fabiana-Madalozzo Coppla, Andrea Freire, Bruna Bittencourt, Ana Armas-Vega, Valeria-Elizabeth-Banderas Benítez, Abraham-Lincoln Calixto, Alessandro-Dourado Loguercio

**Affiliations:** 1 DDS, Ms, PhD, professor, School of Dentistry, Health and Biosciences, Pontifícia Universidade Católica do Paraná, Curitiba, Paraná, Brazil; 2 DDS, Ms, PhD, professor, Department of Restorative Dentistry, School of Dentistry, State University of Ponta Grossa, Ponta Grossa, Paraná, Brazil; 3 DDS, Ms, PhD, Professor Department of Dentistry, Tecnológica Equinoccial University, Quito, Ecuador

## Abstract

**Background:**

Bleaching procedures performed before restorative procedures, due to the oxygen released, affects the quality of bonding restorations. The application of an lower-concentrated antioxidant for one-hour or more can reversal the compromised bonding to bleached enamel, but it was not effective according to the bleaching concentrations applied. The aim of the present study was to evaluate simplified protocol of higher-concentrated sodium ascorbate (35%SA) in bond strength values of enamel bleached with 10%, 16%, 22% carbamide peroxide (CP) or 35% hydrogen peroxide (HP).

**Material and Methods:**

Three hundred and forty enamel surfaces of 85 human third molars were used, divided into 17 groups (n=20), according to the following groups: control = no bleaching and no ascorbic acid application; bleaching (CP10%, CP16%, CP22% at-home and HP 35% in-office) and 35%SA application (no application; 35%SA applied twice for 1-min each [SA2×1], twice for 5-min each [SA2×5] and; twice for 10-min each [SA2×10]). After that, adhesive was applied and composite cylinders were made with Filtek Z350 composite. Microshear test was performed in a universal testing machine. BS values were statistically evaluated using ANOVA and Tukey’s and Dunnet’s (against control) tests, with 5% level of significance.

**Results:**

All bleaching concentrations significantly decrease the enamel bond strength results when compared to control group (*p*<0.05). More concentrated PC (PC22% and PH35%) showed lower enamel bond strength results when compared to lower concentrated PC (PC10% and PC16%; *p*<0.05). A significant increase of the enamel bond strength results were only observed when SA2×5 and SA2×10 were applied (*p*<0.05).

**Conclusions:**

The application of 35% sodium ascorbate for twice 5- and 10-min each was an efficient protocol to reverse the bond strength in bleached enamel at the same level as the no bleaching enamel, independently of the bleaching concentration used.

** Key words:**Tooth bleaching, hydrogen peroxide, sodium ascorbate, bond strength.

## Introduction

Due to the demand for improving dental appearance has grown, tooth bleaching has been an common practice in dental offices. Tooth-bleaching products available in the market are carbamide peroxide (CP) and hydrogen peroxide (HP), which vary according to the concentration of peroxide, application mode, and exposure time. At home-bleaching are usually applied in lower concentrations of HP and CP, on the other side, highly concentrated HP is used for in-office bleaching (1,2).

However, one of the disadvantages of the bleaching procedures is that the oxygen released by HP affects the quality of bonding procedures performed immediately after bleaching. These procedures resulting in decreased bond-strength values, independent of the bleaching agent or technique applied to the tooth structure (3,4). Unfortunately, clinicians need to use adhesive procedures immediately after tooth bleaching in certain cases such as closing diastema, cosmetic recontours, or even in the case of deficient restorations, among others (5).

To overcome this problem, an antioxidant can be applied before the clinician performs the application of the adhesive system. In the majority of the studies, sodium ascorbate (SA) was applied due to higher antioxidant activity of SA in comparison to several evaluated substances (6-10). However, several previously published studies have shown that 10% SA was not suitable when high concentrations of HP or CP were used for bleaching (7,11,12). In this circumstance, the final bond strength to enamel significantly increased when 10% SA was applied. Unfortunately, the final bond strength it would not return to the unbleached controls’ original values (7,12).

Recently, Freire *et al.* (13) reported a direct relationship between the amounts of HP and the amounts of SA. In other words, 35% of SA can effectively reduce higher concentration of HP (35%). This study also showed that the oxidant–antioxidant reaction’s kinetics occur quickly. It means that, it is only necessary a short application time of 35% of SA to produce a satisfactory effect, when higher concentrated HP was used for tooth bleaching (13).

Based on these studies, researchers have recently published studies evaluating the application of higher concentrations of SA (20-35%) on the reversal of the compromised bonding in bleached enamel. Although positive results were found, researchers have not reached consensus regarding the proper time to apply SA because the variability (ranging from 2 to 120 min) of the application times previously evaluated in the literature (14-17).

Therefore, the objective of the present study was to evaluate application protocols for 35% SA in terms of the bond-strength values of enamel that had been bleached in several concentrations. The null hypotheses tested were that the various bleaching gels’ concentrations would not influence the bond strength and that the protocol of 35% SA application would not influence the bond strength after bleaching.

## Material and Methods

-Tooth selection and specimen preparation

For this study, 85 human third molars were obtained after this research had received approval from the Ethics Committee of the Local University (protocol no. 159.520). The enamel surfaces were flattened on wet, 180-grit silicon carbide paper (AROTEC; Cotia, SP, Brazil). The roots of all teeth were removed approximately 2 mm below the cement-enamel junction using a high-speed diamond bur (no. 4138, KG Sorensen; Barueri, SP, Brazil) under constant water irrigation. The dental crowns were then sectioned into diagonals across the teeth’s long axes to produce four enamel specimens (buccal, lingual, and both proximal faces), each with a fragment (cut with a Isomet low-speed saw [South Bay Technology Inc. Buehler, Lake Bluff, IL, USA]).

The specimens were placed on double-sided tape, arranged on glass slides, and embedded in polyvinyl chloride (PVC) tubes (Tigre; São Paulo, SP, Brazil) using acrylic resin (Jet, Artigos Odontológicos Clássico, SP, Brazil). Care was taken to place the exposed enamel surfaces parallel to the PVC tubes’ exteriors. These surfaces were further polished using wet 400- and 600-grit silicon carbide paper for 60 s and then ultrasonically cleaned in distilled water for 5 min.

-Experimental design 

The specimens were randomly divided into 17 groups (n = 20). In addition to the control with no bleaching and no sodium ascorbate (SA) application, the groups varied based on the bleaching procedure (10% CP [Whiteness Perfect 10%, FGM Prod. Odont. Ltda, Joinville, SC, Brazil]; 16% CP [Whiteness Perfect 16%, FGM Prod. Odont. Ltda, Joinville, SC, Brazil]; 22% CP [Whiteness Perfect 16%, FGM Prod. Odont. Ltda, Joinville, SC, Brazil]; 35% HP [Whiteness HP Maxx, FGM Prod. Odont. Ltda, Joinville, SC, Brazil]), on whether the SA treatment (35% SA, Amanda Manipulações Farmacêuticas, Ponta Grossa, PR, Brazil) was applied, and on the SA application time (SA2×10 = SA applied twice for 10 min each, SA2×5 = SA applied twice for 5 min each, and SA2×1 = SA applied twice for 1 min each).

-Bleaching procedures

All bleaching gels were applied according to the respective manufacturers’ instructions ( Table 1). For the 10%, 16%, and 22% CP groups, the CP was applied for 2 hr/day for 21 days; for the 35% HP group, HP was applied in three 15-min applications. The in-office bleaching agent was refreshed every 15 min during the 45-min application period. Two sessions of bleaching were performed with a week-long interval. After each bleaching session, the bleaching gels were removed with a spray of air and water for 30 s, and the enamel surfaces were cleaned with water. During and after bleaching sessions, all groups were stored in artificial saliva at 37ºC.

**Table 1:**
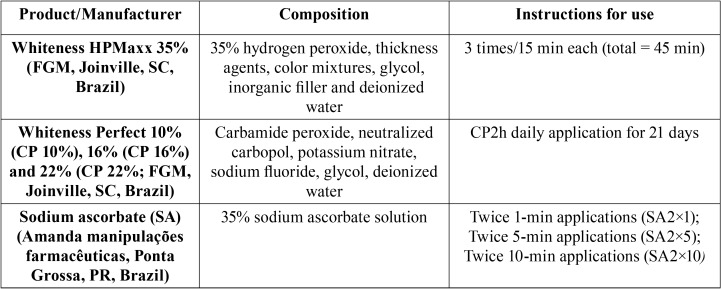
Materials used in the study, composition and respective instructions for use.

-Antioxidant application

Immediately after the end of the bleaching, the bleaching gels were removed with a spray of air and water for 30 s, and the enamel surfaces were cleaned with water. A 35% SA solution was immediately applied according to description provided previously ( Table 1). Another 5 ml of the SA solution was applied and left undisturbed for each application. After that, the enamel surfaces were cleaned with water, and a refresh solution was applied again. At the end of these procedures, the enamel surfaces were cleaned with water.

-Adhesive procedure

For the adhesive procedure, the enamel surfaces were delimited with double-sided tape positioned on the surfaces. The tape was perforated to delimit the adhesive area.18 The delimited areas were etched with 37% phosphoric acid (CondAc 37, FGM, Joinville, SC, Brazil) for 30 s, then rinsed and dried for the same time. The adhesive system (Adper Scotchbond Multi-Purpose; 3M ESPE, St. Paul, MN, USA) was applied according to the manufacturer’s instructions and light-cured for 10 s at 1000 mW/cm2 (Radii-cal, SDI, Victoria, Australia).

Microshear test

After the application of the adhesive system, four polyethylene transparent Tygon tubes (Tygon Medical Tubing Formulations 54-HL, Saint Gobain Performance Plastics, Akron, OH, USA) with the same internal perforation diameter (0.75 mm) and a height of 1 mm were positioned over the double-sided tape, ensuring that their lumens coincided with the circular areas exposed by the perforations. The tubes were then nanofilled with composite resin (Filtek Z350, 3M ESPE; St. Paul, MN, USA), and the resin tube set was positioned over the double-sided tape (with the lumen again coincident with the perforation) and photoactivated for 40 s each (Radii-cal, SDI, Victoria, Australia).

The specimens were stored in distilled water at 37°C for 24 hr, after which the Tygon tubes and double-sided tape were removed with a no. 15 scalpel, exposing only the composite cylinders. A universal testing machine (Kratos; São Paulo, SP, Brazil) was used for the microshear bond test. Each PVC tube containing the bonded specimens was attached to a special shear-testing device (Odeme Biotechnology, Joaçaba, SC, Brazil) and placed in the universal testing machine. A blade was positioned as close as possible to the resin–enamel interface, and shear force was applied to each specimen at a crosshead speed of 1.0 mm/min until failure occurred. The force required to produce failure was then divided by the bonded area, and the bond-strength values were expressed in megaPascals (MPa). After the microshear test, the fractured specimens were examined under a microscope (Olympus-BX 51; Olympus, Tokyo, Japan) at 40× magnification and classified as follows.

• Type 1: adhesive failure.

• Type 2: cohesive failure within the composite;

• Type 3: cohesive failure within the enamel (if the fracture occurred exclusively in the enamel); and

• Type 4: mixed failure (if the fracture involved two types).

Representative images of each failure mode were sputter coated with gold and examined using Scanning Electron Microscopy (Shimadzu, Kyoto, Japan), operated at 15 kV.

-Statistical analysis

As the experimental unit in this study comprised the enamel specimens, all bond-strength specimens with adhesive or mixed failures from the same enamel group were averaged for statistical purposes.

The data were first analyzed using the Kolmogorov-Smirnov test to assess whether the data followed a normal distribution, as well as Barlett’s test for equality of variances to determine if the assumption of equal variances was valid. After confirming the normality of the data distribution and the equality of the variances, the bond-strength data (in MPa) were subjected to statistical analysis.

Two statistical analysis were performed: one using two-way ANOVA (bleaching procedures vs. SA treatment) and Tukey’s post hoc test, and another using ANOVA and Dunnet’s post hoc test. These analyses were meant to compare the enamel bond-strength values obtained in all the experimental groups with those of the control group. For all tests, a 5% significance level was used.

## Results

 Table 2 shows the percentages of the fracture modes for all groups. No cohesive fractures were found in the enamel or in the composite. Adhesive failures had the highest percentage of failures in all groups, independent of the bleaching procedure or the SA treatment.

**Table 2:**
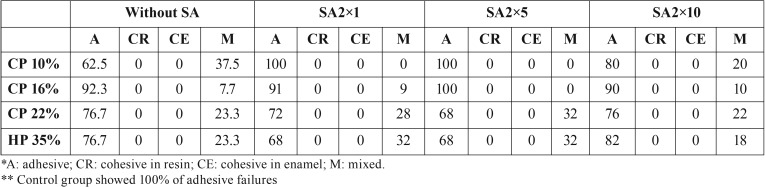
Percentage of fracture modes (*) in each group (**).

All enamel bond-strength values (in MPa) are shown in  Table 3. The two-way ANOVA showed that the cross-product interaction between the two factors (bleaching procedures vs. SA treatment) was statistically significant ( Table 3; *p* = 0.002). For all groups, the bleaching procedures significantly decreased the enamel bond-strength results, regardless of HP concentration, when compared to the control group (no bleaching;  Table 3; *p* < 0.05). In most cases, the 10% and 16% CP groups showed similar bond-strength results; both had higher strengths than the 22% CP and 35% HP groups ( Table 3; *p* < 0.05).

**Table 3:**
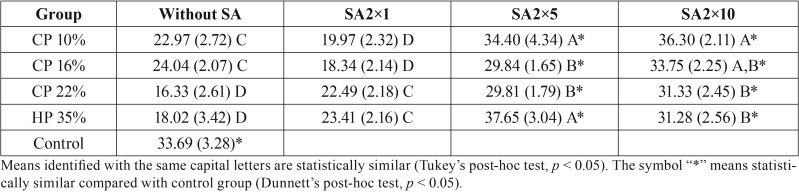
Mean microshear bond strength values (MPa) and the respective standard deviations (±SD), according to experimental groups (*).

However, when 35% SA was used, the results were dependent on the protocol applied. When a lower application time was evaluated (SA2×1), no significant increase in the enamel bond-strength values was observed when compared to the control group ( Table 3; *p* > 0.05). On the other hand, a significant increase in the enamel bond-strength results was observed when the SA was applied twice in 5-min or 10-min intervals (SA2×5 and SA2×10;  Table 3; *p* < 0.05). The bond-strength results for SA2×5 and SA2×10, in all bleaching concentrations, were similar to those of the control group ( Table 3; *p* > 0.05).

## Discussion

The results of the present study show that, regardless of the bleaching concentrations used, all experimental groups experienced a reduction in enamel bond strength when the adhesive restorations were performed immediately after the bleaching procedure. These results confirming the negative effect of bleaching therapies on the bond strength to enamel, as well as, several authors had previously observed (3,4).

Although various theories have been proposed to explain these adverse effects of bleaching procedures, the most accepted theory relates to the presence of residual oxygen (peroxides) on the enamel surface after bleaching (9), as this interferes with oxygen’s polymerization of resinous monomers (19). The release of oxygen from the bleached enamel probably results in incomplete polymerization of the adhesive in these regions (3,4,19). Thus, a reasonable hypothesis is that higher concentrations of bleaching gel, as higher concentration of HP applied in the in-office procedures, lead to greater amounts of available oxygen—and thus, lower bond strengths to enamel, as observed in the present study and in agreement with the previous one (3) Thus, the first null hypothesis should be rejected.

One way to overcome this disadvantage that has been suggested in several previous studies is to establish a wait time between the bleaching and adhesive procedures (4,20). This means that the bonding procedure needs to be done after 7- to 14-day or more after the bleaching to achieve better bonding to enamel (4,20,21).

An interesting option for completing the bonding procedure immediately after the bleaching is to apply an antioxidant, as previously mentioned in the introduction section (6-9). However, a long application time (60 to 120 min) of SA is usually indicated to promote the total removal of oxygen and to allow the reversal of compromised bonding in bleached enamel at the same level of no bleaching enamel (control enamel) (6-9).

The residual effect of HP may extend the bleaching effect and influence the final tooth shade, thus compromising the aesthetic resolution of the restoration when it is carried out immediately after dental bleaching. Thus, to eliminate all peroxide from the teeth, a waiting time is required; this time varies from 7- to 14-day according to the literature (4,20,21).

Thus, the present study proposes a simplified application protocol of 35% SA as a strategy to reverse the compromised bond strength of enamel after bleaching. Although various results were found for the protocols evaluated in this study. The most important bond strength results showed that, the SA2×5 and SA2×10 protocols were similar to the control, regardless of the bleaching concentration. This leads to the rejection of the second null hypothesis.

The main result of the present study should be attributed to a higher concentration of applied SA, which, facilitates faster peroxide elimination (22). The 35% SA used in this study was sufficient for all bleaching procedures, unlike the 10% or 20% SA, which, in previous studies (23,24) have shown satisfactory results only when at-home bleaching was carried out.

Unfortunately, even when using a higher amount of SA, the SA2×1 protocol was not enough to reverse the initially lower bond strength results. An in vitro study showed that, the kinetic reaction between HP and SA revealed that a 5-min length was sufficient to produce an antioxidant effect (13). This means that 5 min of SA application is the minimum time to produce a sufficient antioxidant effect. This could be established by increasing the number of applications of SA, as well as previous observed (22). However, the antioxidant amount and contact time are more significant than the application time (22).

One suggestion based on these results is that the application time and antioxidant amounts are responsible for the positive action of 35% SA, particularly in the SA2×5 and SA2×10 groups. Obviously, the application time is more interesting for clinicians, as Hansen *et al.* (25) previously showed in a bond strength evaluation to dentin. Future studies need to be done to evaluate simpler protocols for applying SA in terms of bleached-enamel bond-strength results.

## Conclusions

The application of bleaching agents to enamel in varying concentrations reduced the enamel’s bond strength, and this decrease was proportional to the concentration of the bleaching agent. However, the dual applications of 35% SA in 5- or 10-min intervals was found to reverse the bond strength losses in bleached enamel, reaching the same level as unbleached enamel.
